# *CTNNB1* mutations are clonal in adamantinomatous craniopharyngioma

**DOI:** 10.1111/nan.12613

**Published:** 2020-04-02

**Authors:** J. R. Apps, C. Stache, J. M. Gonzalez‐Meljem, A. Gutteridge, J. Chalker, T. S. Jacques, T. Forshew, A. Hölsken, J. P. Martinez‐Barbera

**Affiliations:** ^1^ Developmental Biology and Cancer Research and Teaching Department Birth Defects Research Centre UCL Great Ormond Street Institute of Child Health University College London London UK; ^2^ Cancer Research UK Clinical Trials Unit University of Birmingham Birmingham UK; ^3^ Birmingham Women’s and Children’s Hospital NHS Foundation Trust Birmingham UK; ^4^ Bioengineering Department School of Engineering and Science Tecnologico de Monterrey Monterrey Mexico; ^5^ UCL Cancer Institute University College London London UK; ^6^ Great Ormond Street Hospital for Children NHS Foundation Trust London UK; ^7^ Department of Pathology REGIOMED‐Klinikum Coburg Coburg Germany

Adamantinomatous craniopharyngiomas (ACPs) are tumours of the sellar region and although histologically benign, they frequently invade the hypothalamus, visual tracts and local vascular structures. This aggressive behaviour can result in profound chronic morbidity, poor quality of life and increased mortality in long‐term follow‐up [1].

ACPs are histologically complex tumours with variable cystic, calcified and solid components often surrounded by a florid glial and inflammatory reactive tissue. Within tumour epithelia, a peripheral layer of palisading epithelium (PE) encloses more loosely packed stellate cells (i.e. stellate reticulum (SR)) and epithelial whorls [2]. Since the first identification of activating *CTNNB1* mutations in ACP [3], several cohorts have recapitulated this finding and mutations have been identified anywhere between 39 and 100% of the tumours. Nuclear accumulation of β‐catenin and immunohistochemical evidence of activation of the WNT pathway (e.g. *Axin2* expression) is limited to only a small proportion of cells, which in many cases correlate with epithelial whorls (from now on referred to as clusters) [1, 2, 4].

Several studies have investigated the distribution of *CTNNB1* mutations within the different tumour cell compartments. Using Sanger sequencing mutations were identified in both epithelial and ‘mesenchymal’ components [3]. However, Kato et al. did not identify mutations outside of the tumour epithelia [5]. More recently, using laser capture microdissection (LCM) and single‐strand conformational polymorphism analysis, *CTNNB1* mutations were identified in all the eight cases [6]. Surprisingly, two cases harboured more than one *CTNNB1* mutations and in four cases, different *CTNNB1* mutations were found in β‐catenin‐accumulating and non‐accumulating tumour cells [6]. Subsequent studies have failed to identify ACP tumours harbouring more than one *CTNNB1* mutation and only one report has described ACP with coexistence of *CTNNB1* and *BRAF‐V600E* mutations [7]. Genetic tracing experiments in genetically engineered mouse models of ACP have revealed a non‐cell autonomous mechanism of pathogenesis, whereby not all tumour cells contain *CTNNB1* mutations [1]. Together, these findings have brought into question whether *CTNNB1* mutations are present in all ACP tumour cells or only in those accumulating nuclear β‐catenin.

To further investigate the occurrence and cell distribution of *CTNNB1* and *BRAF* mutations in ACPs, we assessed the mutational status of the *CTNNB1* exon 3 and the *BRAF V600E locus*, as well as other commonly mutated brain tumour genes including *H3F3A, HIST1H3B, IDH1* and *IDH2* in 22 tumours [8]. In addition to Sanger sequencing of exon 3 of *CTNNB1*, we used a highly sensitive next generation targeted amplicon sequencing panel (TAm‐Seq) [8]. Compared with conventional targeted sequencing TAm‐Seq replicate amplification of the regions of interest followed by barcoding of separate replicates and deep sequencing, enabling the identification of mutations at variant allele frequencies well below those reliably detected by Sanger sequencing (limited to ~20%) [9]. In three cases, in which cryopreserved tumour tissue was available, we performed LCM using the Zeiss PALM MicroBeam system. We analysed by TAm‐Seq specific tumour cell compartments including the clusters (C), PE and SR as well as local reactive glial tissue as previously described [10](Appendix S1). To confirm the sequencing results, we used immunofluorescence staining using antibodies against specific *CTNNB1* mutations (Appendix S1).

Sanger sequencing analysis was successful in 19 of 22 cases of ACP, but confirmed the presence of a *CTNNB1* mutation only in 12 of 19 tumours (Table 1). In contrast, TAm‐Seq identified *CTNNB1* mutations in all cases (22 of 22), with mutation allele frequencies varying from 3 to 47% (Table 1). The higher detection rate reflects a greater sensitivity of the TAm‐Seq method relative to Sanger sequencing in the identification of mutations with lower allele frequency.

**Table 1:** *CTNNB1* exon 3 sequencing analysis of DNA from 22 cases of ACP

Case No:	Age at diagnosis (years)*	Tumour content (% nuclear area)	DNA conc (copies/ul)	*CTNNB1* mutation by Tam‐Seq	*CTNNB1* mutation by Sanger Seq	Average mutation allele frequency
1	8	20	1100	T41I	T41I	17%
2	14	70	2315	T41I	T41I	28%
3	6	70	390	S37F	No mutation	15%
4	14	60	1155	S45F	Failed**	35%
5	8	10	525	S33F	No mutation	3%
6	12	50	680	S37A	No mutation	16%
7	46	70	770	T41I	No mutation	21%
8	71	80	8854	T41I	T41I	35%
9	adult	80	395	S33C	S33C	39%
10	adult	80	2273	T41I	T41I	29%
11	60	70	143	T41I	T41I	15%
12	83	45	2087	S33C	S33C	19%
13	22	80	439	T41I	T41I	26%
14	87	90	1573	D32N	D32N	31%
15	61	50	195	S33C	S33C	30%
16	65	40	237	T41A	T41A	25%
17	53	40	520	S37A	S37A	17%
18	adult	90	470	I35S	Failed**	43%
19	adult	60	260	S33F	No mutation	25%
20	adult	25	1256	S37F	No mutation	3%
21	adult	20	1460	S33F	No mutation	18%
22	adult	70	4505	S37C	Failed**	47%

* Age not available in all patients.

** Sanger sequencing reaction failed to give readable trace.

All *CTNNB1* mutations have previously been described in ACP, that is D32, S33, I35, S37 and T41 substitutions, which are expected to prevent phosphorylation and therefore disrupt the degradation of β‐catenin [1, 4]. No tumours were found to carry more than one *CTNNB1* pathogenic mutation and no mutations in the hotpots of *BRAF, H3F3A, HIST1H3B, IDH1* or *IDH2* were identified in ACP.

The *CTNNB1* mutation allele frequency in ACP correlated with the percentage of tumour epithelia as assessed by nuclear content (*r* = 0.70, *P* = 0.0004; Appendix S1, Table S1; Figure S1). Indeed, the *CTNNB1* mutation allele frequencies observed across the tumour samples were consistent with the presence of heterozygous *CTNNB1* mutations throughout all tumour epithelial cells. For instance, in case 9, the variant allele frequency of the S33C mutation was 39%, consistent with 78% of the cells harbouring a heterozygous mutation and closely correlating with the histological assessment of tumour epithelia of around 80% (Table 1).

The previous results were confirmed by TAm‐Seq analysis of specific tumour cell compartments isolated by LCM (Appendix S1; Figure S2). *CTNNB1* mutation frequencies were 47–55%, which are consistent with heterozygosity within all tumour compartments (clusters, PE, SR) but nearly zero within the surrounding glial tissue (max 0.65%) and germline DNA (available in two of the three cases) (Table S1).

Finally, we used immunofluorescence staining with specific antibodies against the S33F, S37F and T41I substitutions in β‐catenin on cases of ACP (cases: S33F, n = 2; S37F, n = 4; T41I, n = 6; Figure 1). These studies confirmed the expression mutant β‐catenin protein across the tumour epithelium with nuclear accumulation limited to the cell clusters (Figure 1). Negative control immunofluorescence staining on ACP tumours separately known to harbour other *CTNNB1* mutations confirmed the specificity of the antibodies used (Figure 1) [10].

**Figure 1:**
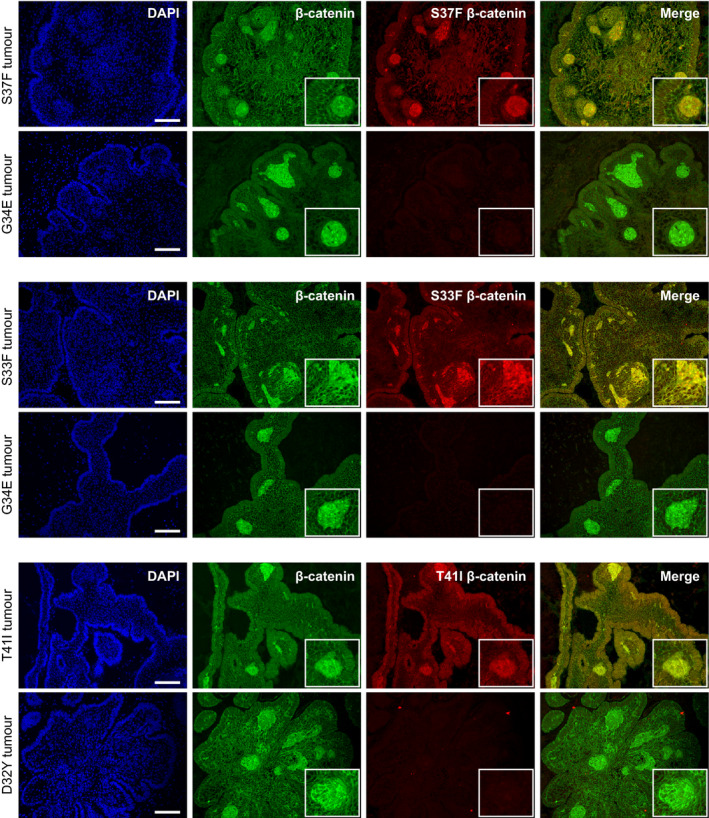
Mutant β‐catenin is expressed throughout the tumour epithelia. Double immunofluorescence stainings on FFPE histological section of ACP tumours harbouring different mutations (shown on the left) using specific antibodies against either S37F, S33F or T41I mutant β‐catenin (red) as well as mutation non‐specific β‐catenin antibody (shown within the panels). Boxes highlight nuclear accumulation within clusters. Scale bar = 100 µm.

In conclusion, we confirm a high prevalence of *CTNNB1* mutations in ACP. The high sensitivity of TAm‐Seq has helped identify *CTNNB1* mutations in all ACP samples analysed, including those with very low allele frequencies. This suggests that failure to identify *CTNNB1* mutations in a low proportion of ACP tumours in previous studies using Sanger sequencing, single‐strand conformation polymorphism analysis, exome sequencing and targeted next generation sequencing may reflect methodological limitations.

We reveal that all cellular components of the tumour epithelium harbour the same *CTNNB1* mutation and found no evidence of second *CTNNB1* mutations, even at low allele frequencies. Additionally, we show the presence of *CTNNB1* mutations throughout the tumour epithelia, which is consistent with the early stages of tumourigenesis in the embryonic mouse model of ACP. In this model, oncogenic *Ctnnb1* mutation is present throughout the entire tumoural pituitary, but nuclear accumulation of β‐catenin is limited to only a small proportion of cells mostly forming clusters, which are molecularly analogous to the human clusters [11, 12]. The mechanism behind why β‐catenin is accumulated only in single cells or clusters despite the presence of *CTNNB1* mutations in other epithelial tumour cells (i.e. palisading epithelium and stellate reticulum) is not known.

## Ethics

Experiments were performed under NHS Research Ethics Committee approval (14/LO/2265) or approval from individual biobanks. Where required, informed consent was obtained from all individual participants included in the study.

## Funding

Funding for this research was provided by Cancer Research UK, the Children’s Cancer and Leukaemia Group, Children with Cancer UK (15/190), MRC (MR/M125/1), the Brain Tumour Charity (SIGNAL and EVEREST), Great Ormond Street Hospital Children’s Charity and the National Institute of Health Research Biomedical Research Centre at Great Ormond Street Hospital for Children NHS Foundation Trust and University College London. J.R.A. is supported by a Cancer Research UK Clinical Trials Fellow. JMGM is part of the Translational OMICS Strategic Focus Group at Tecnologico de Monterrey. J.P.M.‐B. is a Great Ormond Street Hospital for Children’s Charity Principal Investigator.

## Conflict of Interest

The Editors of *Neuropathology and Applied Neurobiology* are committed to peer‐review integrity and upholding the highest standards of review. As such, this article was peer‐reviewed by independent, anonymous expert referees, and the authors (including TSJ and JPMB) had no role in either the editorial decision or the handling of the paper.

## Supporting information


**Figure S1** **.** A. Correlation between mutation allele frequency and histologically assessed tumour content (%nuclei). Arrows indicate cases 9 and 17. B. Examples of ACP FFPE histological sections stained with haematoxylin and eosin.

**Figure S2** **.** Representative images of laser capture microdissection (LCM): A. Areas selected for LCM are highlighted by colours: Clusters (green), stellate reticulum (black), palisading epithelium (blue), reactive glial tissue (yellow). B. Example showing the excision of a cluster. C. Example of multiple clusters pooled before DNA extraction. Scale bar =100μm.

**Table S1** **.** Laser capture microdissection identifies CTNNB1 mutations in all ACP tumour compartments.

**Appendix S1** **.** Supplementary Methods
