# Supplementary figures and images for: CTNNB1 mutations are clonal in adamantinomatous craniopharyngioma
Source: Neuropathol Appl Neurobiol. 2020 Apr 2;46(5):510–4. doi: 10.1111/nan.12613 (PMC7610141; doi:10.1111/nan.12613)

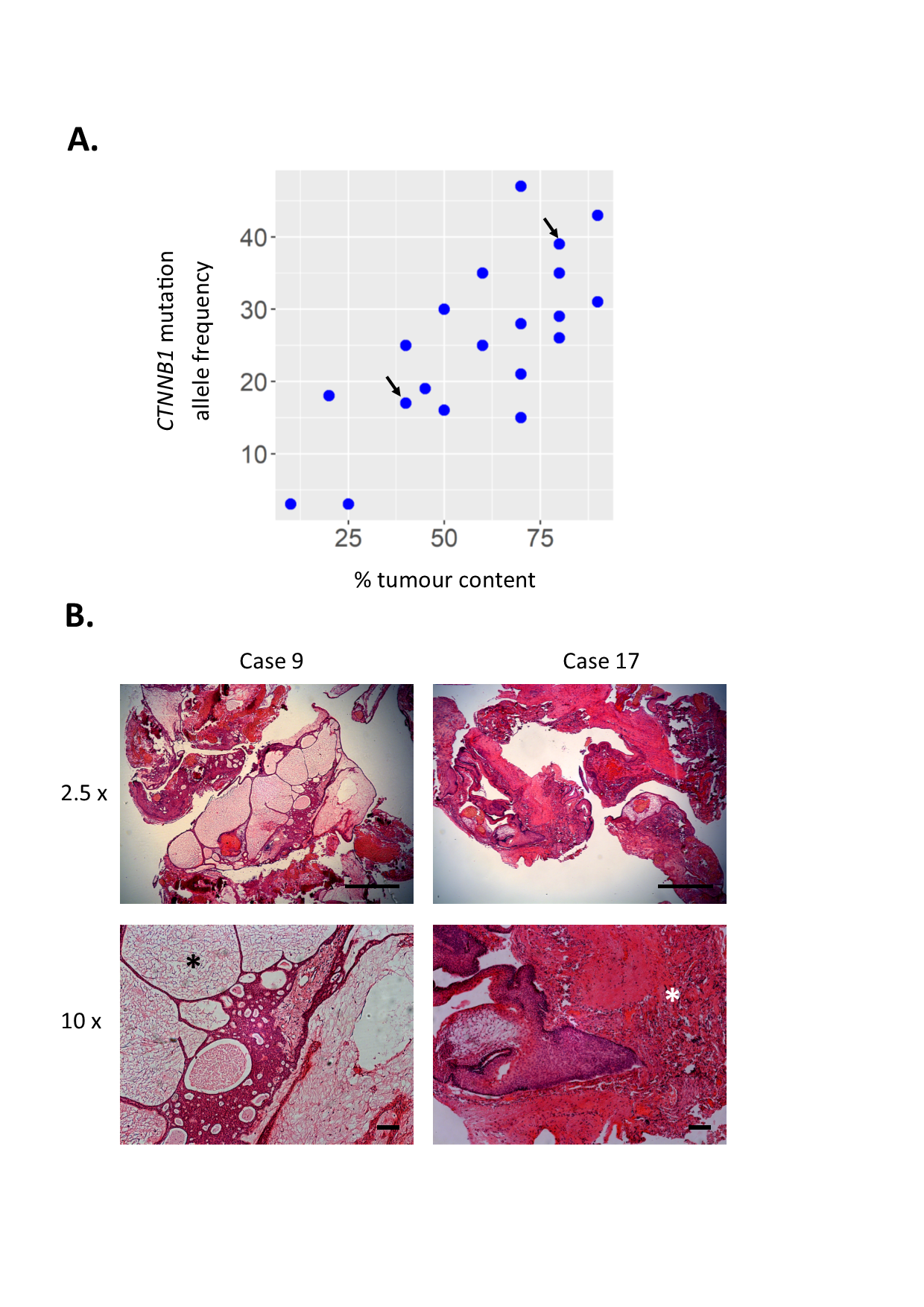

Supplement: Supplementary file 1 — Figure S1 . A. Correlation between mutation allele frequency and histologically assessed tumour content (%nuclei). Arrows indicate cases 9 and 17. B. Examples of ACP FFPE histological sections stained with haematoxylin and eosin. [file NAN-46-510-s001.tif]

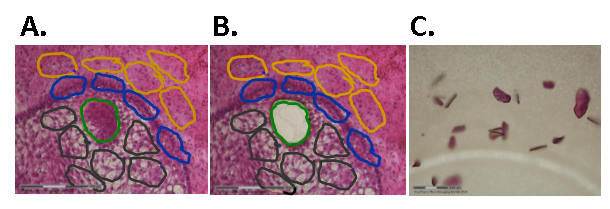

Supplement: Supplementary file 2 — Figure S2 . Representative images of laser capture microdissection (LCM): A. Areas selected for LCM are highlighted by colours: Clusters (green), stellate reticulum (black), palisading epithelium (blue), reactive glial tissue (yellow). B. Example showing the excision of a cluster. C. Example of multiple clusters pooled before DNA extraction. Scale bar =100μm. [file NAN-46-510-s002.tif]
