# Supplementary material for: CTNNB1 mutations are clonal in adamantinomatous craniopharyngioma
Source: Neuropathol Appl Neurobiol. 2020 Apr 2;46(5):510–4. doi: 10.1111/nan.12613 (PMC7610141; doi:10.1111/nan.12613)
Supplement: Supplementary file 3 — Table S1 . Laser capture microdissection identifies CTNNB1 mutations in all ACP tumour compartments. [file NAN-46-510-s003.docx]

**Supplementary Table 1: Laser Capture Microdissection identifies *CTNNB1* mutations in all ACP tumour compartments in three cases of ACP.**

| Mutation | Mutation allele frequency | | | | |
| --- | --- | --- | --- | --- | --- |
|  | Clusters | Palisading epithelium | Stellate reticulum | Glial tissue | Germline |
| CTNNB1-S37F | 49% | 47% | 49% | 0% | 0% |
| CTNNB1-G34E | 49% | 49% | 51% | 0% | N/A |
| CTNNB1-G34E | 52% | 54% | 55% | 0.65% | 0% |
