# Supplementary material for: CTNNB1 mutations are clonal in adamantinomatous craniopharyngioma
Source: Neuropathol Appl Neurobiol. 2020 Apr 2;46(5):510–4. doi: 10.1111/nan.12613 (PMC7610141; doi:10.1111/nan.12613)
Supplement: Supplementary file 4 — Appendix S1 . Supplementary Methods [file NAN-46-510-s004.docx]

**Supplementary Methods**

**Tumour content estimation:** As per Apps *et al*,, 2018, Tumour content was estimated as the proportion of tumour cell nuclei within the whole of the frozen section (i.e. including glial reactive tissue). Cell nuclei, rather than area were counted to compensate for the lower cell density of the host-derived glial reactive tissue relative to the tumour. This was performed by TSJ alongside JRA prior to sequencing analysis.

**Sequencing.** Targeted amplicon sequencing (TAm-Seq) was performed as per Stone *et al*., using the method developed in Weaver *et al. (1, 2)*.

For Sanger Sequencing, a 415bp PCR product was amplified using Amplitaq Gold (Applied Biosystems) with specific primers in a touchdown programme (see below). Amplicons were sequenced using BigDye Terminator kit (Applied Biosystems) and an Applied Biosystem 3500 analyser.

PCR primer sequences for sanger sequencing of CTNNB1

| β-Catenin F | CATTTCCAATCTACTAATGCT |
| --- | --- |
| β-Catenin R | CTGCATTCTGACTTTCAGTAA |

PCR Protocol for sanger sequencing of CTNNB1

| Touch Down Cycle - TD45 | |  |
| --- | --- | --- |
| 95^o^C | | 7mins |
| 93^o^C | x10 (<1^o^C per cycle) | 45secs |
| 65 ^o^C → 56 ^o^C |  | 45secs |
| 72^o^C |  | 1min 30secs |
| 93^o^C | x35 | 45secs |
| 56^o^C |  | 45secs |
| 72^o^C |  | 1min 30secs |
| 72^o^C | | 10mins |
| 4 ^o^C | | Forever |

**Laser capture microdissection (LCM):** LCM of ACP was performed as previously described using a separate cohort of cases of frozen ACP tissue (3). For LCM samples, DNA was extracted using the Qiagen QIAmp DNA Microkit. DNA was quantified by digital droplet PCR (ddPCR) and underwent TAm-Seq as above (2, 4).

**Immunofluorescence:** Immunofluorescence was performed as previously described (3). The specific antibodies and concentrations are shown below.

| Name (clone/catalog no) | Species | Supplier | Antigen retrieval | Concentration | Secondary Antibody or Amplification |
| --- | --- | --- | --- | --- | --- |
| CTNNB1 RB-9035-P1 | Rabbit | Thermofisher | Tris-EDTA pH9 | 1:300 |  |
| CTNNB1 S37F mutation specific antibody (26168) | Mouse | Neweast Biosciences | Tris-EDTA pH9 | 1:25 | Anti-Mouse biotinylated |
| CTNNB1 S33F mutation specific antibody (26304) | Mouse | Neweast Biosciences | Tris-EDTA pH9 | 1:50 | Anti-Mouse biotinylated |
| CTNNB1 T41 mutation specific antibody (26306) | Mouse | Neweast Biosciences | Tris-EDTA pH9 | 1:25 | Anti-Mouse biotinylated |

1. Stone TJ, Keeley A, Virasami A, Harkness W, Tisdall M, Izquierdo Delgado E, et al. Comprehensive molecular characterisation of epilepsy-associated glioneuronal tumours. Acta Neuropathol. 2018;135(1):115-29.

2. Weaver JMJ, Ross-Innes CS, Shannon N, Lynch AG, Forshew T, Barbera M, et al. Ordering of mutations in preinvasive disease stages of esophageal carcinogenesis. Nat Genet. 2014;46(8):837-43.

3. Apps JR, Carreno G, Gonzalez-Meljem JM, Haston S, Guiho R, Cooper JE, et al. Tumour compartment transcriptomics demonstrates the activation of inflammatory and odontogenic programmes in human adamantinomatous craniopharyngioma and identifies the MAPK/ERK pathway as a novel therapeutic target. Acta Neuropathol. 2018;135(5):757-77.

4. Hindson BJ, Ness KD, Masquelier DA, Belgrader P, Heredia NJ, Makarewicz AJ, et al. High-throughput droplet digital PCR system for absolute quantitation of DNA copy number. Analytical chemistry. 2011;83(22):8604-10.
